# *Cryptosporidium* spp., *Giardia duodenalis*, *Enterocytozoon bieneusi* and Other Intestinal Parasites in Young Children in Lobata Province, Democratic Republic of São Tomé and Principe

**DOI:** 10.1371/journal.pone.0097708

**Published:** 2014-05-20

**Authors:** Maria Luísa Lobo, João Augusto, Francisco Antunes, José Ceita, Lihua Xiao, Vera Codices, Olga Matos

**Affiliations:** 1 Unidade de Parasitologia Médica, Grupo de Protozoários Oportunistas/VIH e Outros Protozoários, CMDT, Instituto de Higiene e Medicina Tropical, Universidade Nova de Lisboa, Lisboa, Portugal; 2 Centro Hospitalar do Algarve, Hospital de Portimão, Portimão, Portugal; 3 Faculdade de Medicina, Universidade de Lisboa, Lisboa, Portugal; 4 Hospital Aires de Menezes, São Tomé, São Tomé e Príncipe; 5 Division of Foodborne, Waterborne and Environmental Diseases, Centers for Disease Control and Prevention, Atlanta, Georgia, United States of America; UCSD, United States of America

## Abstract

Rare systemic studies concerning prevalence of intestinal parasites in children have been conducted in the second smallest country in Africa, the Democratic Republic of São Tomé and Príncipe. Fecal specimens from 348 children (214 in-hospital attending the Aires de Menezes Hospital and 134 from Agostinho Neto village) in São Tome Island were studied by parasitological and molecular methods. Of the 134 children from Agostinho Neto, 52.2% presented intestinal parasites. 32.1% and 20.2% of these children had monoparasitism and polyparasitism, respectively. *Ascaris lumbricoides* (27.6%), *G. duodenalis* (7.5%), *T. trichiura* (4.5%) and *Entamoeba coli* (10.5%) were the more frequent species identified in the children of this village. *Giardia duodenalis* (7.5%) and *E. bieneusi* (5.2%) were identified by PCR. Nested-PCR targeting *G. duodenalis* TPI identified Assemblage A (60%) and Assemblage B (40%). The *E. bieneusi* ITS-based sequence identified genotypes K (57.1%), KIN1 (28.6%) and KIN3 (14.3%). Among the 214 in-hospital children, 29.4% presented intestinal parasites. In 22.4% and 7.0% of the parasitized children, respectively, one or more species were concurrently detected. By microscopy, *A. lumbricoides* (10.3%) and *Trichiuris trichiura* (6.5%) were the most prevalent species among these children, and *Cryptosporidium* was detected by PCR in 8.9% of children. GP60 locus analysis identified 6.5% of *C. hominis* (subtypes IaA27R3 [35.7%], IaA23R3 [14.3%], IeA11G3T3 [28.6%] and IeA11G3T3R1 [21.4%]) and 2.3% of *C. parvum* (subtypes IIaA16G2R1 [20.0%], IIaA15G2R1 [20.0%], IIdA26G1 [40.0%] and IIdA21G1a [20.0%]). *G. duodenalis* and *E. bieneusi* were identified in 0.5% and 8.9% of the in-hospital children, respectively. *G. duodenalis* Assemblage B was characterized. The *E. bieneusi* genotypes K (52.6%), D (26.4%), A (10.5%) and KIN1 (10.5%) were identified. Although further studies are required to clarify the epidemiology of these infectious diseases in this endemic region the significance of the present results highlights that it is crucial to strength surveillance on intestinal pathogens.

## Introduction

Intestinal parasites are an important cause of morbidity and mortality worldwide, especially in low-middle income countries in tropical and sub-tropical regions. The hot and humid climate, high population density, poor conditions of hygiene and the presence of insects as vectors or merely as mechanic carriers of parasites, limited economic resources, and some social-cultural habits (food and others), promote undoubtedly the parasite transmission in these regions [1]. It is estimated that worldwide, about two billion people are affected by soil transmitted helminths such as *Ascaris lumbricoides*, *Ancylostoma*/*Necator* spp. and *Trichuris trichiura*, 50 million by *Entamoeba histolytica* and 2.8 million by *Giardia duodenalis* (synonymous *G. lamblia* and *G. intestinalis*) [2]–[4]. Another protozoan, *Cryptosporidium,* is considered an important agent of intestinal disease in humans worldwide [5], [6]. More recently, the emerging microsporidia, especially *Enterocytozoon bieneusi* species has often been described as a frequent human pathogenic microorganism causing gastrointestinal infections and/or disseminated pathology, according to the species involved [7]. In addition to infecting humans, *G. duodenalis*, *Cryptosporidium* spp, and microsporidia are found in a wide range of animals including livestock, companion animals, and wildlife worldwide.

Despite their wide occurrence, giardiasis and cryptosporidiosis are considered neglected diseases by the World Health Organization, largely due to lack of studies in low-middle income countries [8]. The epidemiology of microsporidiosis is less clear in these deprived regions where few studies have been carried out.

The transmission to humans of some helminthes, protozoa and microsporidia is via the fecal-oral route and can occur through direct contact with infected persons (anthroponotic transmission) or animals (zoonotic transmission), or by ingestion of contaminated food (foodborne transmission) or water (waterborne transmission) [9]. The fecal-oral transmission route is facilitated by the contamination of water and soil due to the absence of adequate sanitation and hygiene, especially in rural areas of developing countries. When the water/soil is contaminated, the resilient infective forms (eggs, cysts, spores) of the pathogenic organisms can be transported to vegetables, fruit, hands, tools, handles doors, currency, etc. and be easily swallowed accidentally by humans [10].

The use of molecular approaches has allowed inter-and intraspecific genetic characterization of *G. duodenalis*, *Cryptosporidium* spp., and *E. bieneusi,* thus facilitates the identification of infection source(s) of these pathogens and improve understanding of their epidemiology.

With a population of about 183,176 [11], the Democratic Republic of São Tomé and Príncipe (DRSTP) is the second smallest country in Africa, and lies approximately 180 miles from Gabon on the West African coast. The two main islands (São Tomé and Principe) form part of a chain of extinct volcanoes and both are mountainous. São Tomé and Principe islands feature a tropical wet and dry climate with a relatively lengthy wet season (October to May) and a short dry season (June-September). The country is one of the smallest economies in Africa. Economic growth is estimated to be 4.5% in 2012, marking a gradual recovery. Nevertheless, poverty remains rampant affecting more than 50% of the population [12]. Enteric diseases in low-income countries are an important public health problem that may be manifested as impaired mental health, impaired growth and poor academic performance, especially among children and adolescents, which are more at risk of infections than the adult population [13]. São Tomé and Principe islands have all environmental, economic and social conditions that favor the occurrence and transmission of enteric pathogens. However, limited systemic studies concerning prevalence of intestinal parasites in children have been conducted to elucidate the epidemiology of these parasites in DRSTP [14], [15]. The present study was conducted to assess the prevalence of helminthes, protozoan and the microsporidia *Enterocytozoon bieneusi* in children in DRSTP.

## Materials and Methods

### Ethical Statement

This study was approved by the Ministry of Health and Social Affairs of The Democratic Republic of São Tomé e Príncipe and Dr. Ayres de Menezes Hospital (S. Tomé). Informed written consent was obtained from the parents or guardians on the behalf of the children involved in the study.

### Patient Population and Specimen Collection and Processing

Fecal specimens were collected from 348 children in São Tomé Island from July 2009 to September 2009. Among them, 214 in-hospital children attending the Aires de Menezes Hospital (0°21'23"N 6°43'20"E, integrated in an urban area of São Tomé Island) and all children (134) living at Agostinho Neto village (0°22'4"N 6°38'38"E), a typical African rural community with more than 1,600 acres, north District of Lobata (São Tomé Island) were enrolled in the study. Multiple specimens (two to three) were collected from both groups of children. A structured questionnaire was used to collect socio-demographic characteristics (age, gender), clinical information (symptoms: diarrhea, fever and gastrointestinal complaints) and other potential risk factors (source of drinking water, sanitation, feeding habits, food preservation and domestic animals). Diarrhea was defined as the emission of three or more unformed stools within a 24-h period. All samples were stored at 4°C in 2.5% (w/v) potassium dichromate solution without preservatives prior to being used in parasitological and molecular characterizations.

The stool samples were treated by a modified water-ether sedimentation method. For detection of parasitic agents, a wet mount of each fresh concentrate fecal sample was prepared in a saline and iodine solution and observed under light microscopy for eggs, cysts and trophozoites of various parasites. Also, a permanent slide was prepared for each sample. Smears were examined using modified Ziehl–Neelsen staining for the presence of *Cryptosporidium* spp., *Cyclospora* sp. and *Cystoisospora belli*.

### Microscopy and PCR methods

PCR was performed on all collected samples. Genomic DNA was extracted from feces using the FastDNA SPIN kit for soil [16].

DNA preparations were analyzed by a small subunit (SSU) rRNA-based polymerase chain reaction (PCR)-restriction fragment length polymorphism (RFLP) technique that detects and differentiates common *Cryptosporidium* species in humans [17]. *Cryptosporidium hominis* and *Cryptosporidium parvum* present were subtyped by a PCR-sequencing tool targeting a ∼400-bp fragment of the 60 kDa glycoprotein (gp60) gene [18]. The recommended subtype nomenclature was used in naming *C. hominis* and *C. parvum* subtypes [9].

*E. bieneusi* was detected by nested-PCR analysis of the entire internal transcribed spacer (ITS) region of the rRNA gene. *E. bieneusi* genotyping was carried out by sequence analysis of the ITS rRNA gene [7].

A modified nested-PCR protocol [19] targeting the triosephosphate isomerase (tpi) gene of *G. duodenalis* was developed for this study. Genotyping primers specific for *G. duodenalis* were designed using Primer 3.0 software ((http://frodo.wi.mit.edu/cgi-bin/primer3/primer3.cgi) based on the TPI sequence (GeneBank accession no http://www.ncbi.nlm.nih.gov/sites/entrez?db=pubmed). The forward primer TPI-FW1 (5′CAGAAAATAAATIATGCCTGCTC3′) and reverse primer TPI-RV1 (5′CAAACCTTiTCCGCAAACC3′) amplify a 618 bp fragment. The TPI-FW2 (5′ CCCTTCATCGGIGGTAACTTCAA3′) and the TPI-RV2 (5′ACATGGACiTCCTCTGCCTGCTC3′) amplify a 557 bp fragment. Amplification was carried out in 50 µl reactions containing 1× PCR buffer, 2.5 mmol/l MgCl_2_, 200 µmol/l dNTP mix, 100 nM of each primer, 1U Taq DNA polymerase (Bioline), and 0.2 mg/ml bovine serum albumin. Each PCR run had a negative control of ultrapure water and a positive control containing template DNA.

Three PCR replicates per sample using 3 µl of extracted DNA per PCR, were performed to increase the accuracy of the methods. PCR products were analyzed by 1.5% agarose gel electrophoresis and ethidium bromide-staining.

Samples that gave positive results with at least one method used were considered positive for this study.

### DNA sequence analysis

The secondary PCR products were purified using a Jetquick kit and sequenced in both directions on an ABI3100 automated sequencer (Applied Biosystem, Foster City, CA.). The accuracy of the nucleotide sequence was confirmed by sequencing two separate PCR products from the same sample. The sequences obtained were analyzed together with the reference sequences from the GenBank database using the BLASTN (www.ncbi.nlm.nih.gov) and ClustalX (ftp://ftp-igbmc.u-strasbg.fr/pub/ClustalX/) programs to determine *Cryptosporidium*, *G. duodenalis* and *E. bieneusi* genetic variability.

### Data analysis

The Chi-square test or Fisher's exact test was used to compare prevalence of parasites among age groups, gender, symptoms (diarrhea, fever and gastrointestinal complaints) and to evaluate potential risk factors (source of drinking water: piped water, well; sanitation: public sewer, septic tank; indoor toilet; feeding habits: breastfeeding, bottlefeeding, solids, breastfeeding + solid feed, bottle feeding + solid feed, breast + bottle + solids; food preservation: refrigerators; domestic animals). All statistical analyses were performed using SPSS version 17.0. A *P* value of <0.05 was considered statistically significant.

## Results

### 1. Children from a rural community (Agostinho Neto village)

#### 1.1 Helminthes, protozoan and *Enterocytozoon bieneusi*

The socio-demographical and clinical data from children enrolled and the results obtained in the present study are summarized in Tables 1, 2 and 3.

**Table 1:** Socio-demographic and clinical features of children from Agostinho Neto Village and Aires de Menezes Hospital (DRSTP) enrolled in the study.

Characteristics			Agostinho Neto Village (N = 134)		Aires de Menezes Hospital (N = 214)	
			n	%	n	%
**Sex**						
	**Male**		**63**	**47.0**	**123**	**57.5**
	**Female**		**71**	**53.0**	**91**	**42.5**
**Age (years)**						
	**Median**		**4**	**-**	**1**	**-**
	**Range**		**0.167^a^–10**	**-**	**0,027^b^-10**	**-**
**Symptoms**						
	**Diarrhea**	**No**	**116**	**86.6**	**85**	**39.7**
		**Yes**	**18**	**13.4**	**129**	**60.3**
	**Fever**	**No**	**123**	**91.8**	**104**	**48.6**
		**Yes**	**11**	**8.2**	**110**	**51.4**
	**GC**	**No**	**101**	**75.4**	**90**	**42.1**
		**Yes**	**33**	**24.6**	**124**	**57.9**
**Source of drinking water**						
	**Piped water**	No	**108**	**80.6**	**135**	**63.1**
		**Yes**	**26**	**19.4**	**79**	**36.9**
	**Well**	**No**	**106**	**79.1**	**184**	**86.0**
		**Yes**	**28**	**20.9**	**30**	**14.0**
**Sanitation**						
	**Sewage**	**No**	**118**	**88.1**	**142**	**66.4**
		**Yes**	**16**	**11.9**	**72**	**33.6**
	**Septic tank**	**No**	**91**	67.9	151	**70.6**
		**Yes**	**43**	**32.1**	**63**	**29.4**
	**In-door toilet**	**No**	**64**	**47.8**	**98**	**45.8**
		**Yes**	**70**	**52.2**	**116**	**64.2**
**Feeding habits**						
	**Breastfeeding**		**22**	**16.4**	**35**	**16.4**
	**Bottle feeding**		**1**	**0.7**	**2**	**0.9**
	**Solid food**		**111**	**82**	**87**	**40.7**
	**BrF+SF**		**0**	**0**	**54**	**25.2**
	**BoF+SF**		**0**	**0**	**15**	**7.0**
	**BrF+BoF+SF**		**0**	**0**	**21**	**9.8**
**Food preservation**						
	**Fridge**	**No**	**101**	**75.4**	**143**	66.8
		**Yes**	**33**	**24.6**	**71**	**33.2**
**Domestic animals**						
		**No**	**78**	**58.2**	**89**	**41.6**
		**Yes**	**56**	**41.8**	**125**	**58.4**

**Note**: ^a^0.167corresponds to 2 months old; ^b^0.027 corresponds to 10 days old; GC: gastrointestinal complains; BrF+SF: breastfeeding and solid food; BoF+SF: bottle feeding and solid food; BrF+BoF+SF: breastfeeding, bottlefeeding and solid food.

**Table 2:** Distribution of intestinal parasites identified by microscopy and/or PCR among the children enrolled in the study.

	Agostinho Neto Village (N = 134)	Aires de Menezes Hospital (N = 214)
	Microscopy and/or PCR n (%)	Microscopy and/or PCR n (%)
Negative	64 (47.8)	151 (70.6)
Positive	70 (52.2)	63 (29.4)
Monoparasitism	43 (32.1)	48 (22.4)
Polyparasitism	27 (20.1)	15 (7.0)
Helminthes	45 (33.6)	27 (12.6)
Protozoa	39 (29.1)	24 (11.2)
Fungi	7 (5.2)	19 (8.9)

**Table 3:** Distribution of intestinal parasites (microscopy and/or PCR) and genetic characterization of *Cryptosporidium* spp., *Giardia duodenalis* and *Enterocytozoon bieneusi* among children from the two communities analyzed.

	Agostinho Neto Village (N = 134)			Aires de Menezes Hospital (N = 214)		
	Microscopy n (%)	PCR n (%)	Species typing^a^ (n)	Microscopy n (%)	PCR n (%)	Species typing^a^ (n)
**Helminthes**						
***Ascaris lumbricoides***	**37 (27.6)**	ND	ND	**22 (10.3)**	ND	ND
***Ancylostoma*** ** spp.**	**1 (0.8)**	ND	ND	**1 (0.5)**	ND	ND
***Hymenolepis nana***	**2 (1.5)**	ND	ND	**0**	ND	ND
***Hymenolepis*** ** sp.**	**3 (2.2)**	ND	ND	**0**	ND	ND
***Schistosoma intercalatum***	**0**	ND	ND	**2 (0.9)**	ND	ND
***Strongyloides stercoralis***	**1 (0.8)**	ND	ND	**0**	ND	ND
***Trichuris trichiura***	**6 (4.5)**	ND	ND	**14 (6.5)**	ND	ND
**Protozoa**						
***Cryptosporidium*** ** spp.**	**0**	**0**	**0**	**9 (4.2)**	**19 (8.9)**	
***C. hominis***	ND	**0**	**0**	ND	**14 (6.5)**	**IeA11G3T3R1 (3) IeA11G3T3 (4) IaA27R3 (5) IaA23R3 (2)**
***C. parvum***	ND	**0**	**0**	ND	**5 (2.3)**	**IIdA21G1a(1) IIdA26G1 (2) IIaA16G2R1(1) IIaA15G2R1(1)**
***Cyclospora*** ** sp.**	**1 (0.8)**	ND		**0**	ND	
***Entamoeba coli***	**14 (10.5)**	ND		**1 (0.5)**	ND	
***Entamoeba*** ** sp.**	**2 (1.5)**	ND		**0**	ND	
***Giardia duodenalis***	**25 (7.5)**	**10 (7.5)^b^**	**A^c^ (6); B (4)**	**4 (1.9)**	**1 (0.5)^b^**	**B (1)**
**Fungi**						
***Enterocytozoon bieneusi***	ND	**7 (5.2)**	**Type IV(4); Kin1 (2); Kin3 (1)**	ND	**19 (8.9)**	**Type IV (10), D (5), A (2), Kin1 (2)**

**Note**: ^a^Subtypes, Assemblages and genotypes are indicated for *C. hominis* and *C. parvum*, *G. duodenalis* and *E. bieneusi*, respectively; ^b^Samples identified by PCR were also positive by microscopy;^ c^Despite several efforts is was not possible to identified A sub-assemblages; ND – Not determined.

The age of the children varied between 2 months and 10 years old, with a median of 4 years. Among the 134 children included in this study, 53.0% (71/134) were females and 47.0% (63/134) were males (Table 1).

Among the 134 children with complete data, 13.4% (18/134) reported diarrhea, 24.6% (33/134) had gastrointestinal complaints and 8.2% (11/134) reported fever. The primary sources of drinking water were taps (19.4%; 26/134) and wells (20.9%; 28/134). Only 11.9% (16/134) of the children lived in houses with public sewer and 32.1% (43/134) with septic tank. More than half of the children studied (52.2%; 70/134) reported to have indoor toilet.

Regarding to the feeding habits most children were solid diet (82.0%; 111/134), while only 16.4% (22/134) and 0.7% (1/134) were breast fed and bottle fed, respectively. Only a minority of the children 24.6% (33/134) had refrigerators for food preservation. About 41.8% (56/134) of the children had domestic animals in the house.

Of the 134 children analyzed from Agostinho Neto village, 52.2% (70/134) presented intestinal parasites detected by microscopy and/or PCR. 32.1% (43/134) and 20.2% (27/134) of the children had single species infection and polyparasitism, respectively (Table 2).

Of the specimens tested by microscopy, 27.6% (37/134) were positive for *A. lumbricoides*, 7.5% (25/134) for *G. duodenalis*, 4.5% (6/134) for *T. trichiura* and 10.5% (14/134) for *Entamoeba coli*. Other helminthes such as *Ancylostoma* spp. (0.8%; 1/134), *Hymenolepis* sp. (2.2%; 3/134), *Hymenolepis nana* (1.5%; 2/134), *Strongyloides stercoralis* (0.8%; 1/134) and protozoa such as *Entamoeba* sp. (1.5%; 2/134) and *Cyclospora cayetanensis* (0.8%; 1/134) were also identified in the children of this village (Tables 2 and 3).

*Giardia duodenalis* and *E. bieneusi* were identified respectively in 7.5% (10/134) and 5.2% (7/134) of the children by PCR. All the 10 *G. duodenalis* PCR-positive children were also positive by microscopy. Nested-PCR targeting TPI identified Assemblage A (60%; 6/10) and Assemblage B (40%; 4/10) among the 10 *G. duodenalis* positive samples. The *E. bieneusi* ITS-based sequence identified three genotypes previously described: K (57.1%; 4/7), KIN1 (28.6%; 2/7) and KIN3 (14.3%; 1/7).

Among the children positive for helminthes, protozoa and *E. bieneusi* the majority reported no symptoms. Out of a total of 45 helminth-positives, 25 *G. duodenalis*-positive and seven *E. bieneusi*-positive children, only four, two and one child, respectively, reported to have diarrhea. Fourteen children with helminth infection had gastrointestinal complaints and four presented fever. Only seven of the *G. duodenalis*-positive children reported fever.

#### 1.2 Intestinal parasites and risk factors

No statistically significant differences were observed between the presence of the identified parasites in fecal samples among children and the following parameters evaluated: age, gender, symptoms, presence of public sewer, indoor toilet, refrigerators and domestic animals.

The detection of intestinal parasites in children by microscopy was significantly associated with the presence of septic tank next to home. About 72.1% (31/43) of the children having septic tank at home were parasitized, comparing to 41.8% (38/91) of children who used other methods of excreta disposal (χ2 = 10,758, *P* = 0.001). This was the case for both monoparasitism (34.9%;15/43) or polyparasitism (37.2%; 16/43) (χ2 = 15.654, *P*≤0.001).

The identification of *G. duodenalis* by TPI based nested-PCR was more common in children (17.9%; 5/28) who reported having a well next to home than in children (4.7%; 5/106) without well (Fisher's exact test  = 5,538, *P* = 0.033). In addition, the presence of *G. duodenalis* was more frequent in children having septic tank (18.6%; 8/43) near the house than in children who did not have septic tank near home (2.2%) (Fisher's exact test  = 11,383, *P* = 0.002).

Among children who were positive for intestinal parasites by microscopy, 60.7% (68/112) consumed solid food (exclusively or on had bottle feeding) and 4.6% (1/22) were breastfed. The association between the presence of intestinal parasites and children on solid food diet (exclusively or had combined feeding) was highly significant (χ2 = 23,226, *P*≤0.001).

In the group of breastfed, all children had more than 2 years old and only in one child (4.6%) was detected the presence of intestinal parasites by microscopy. On the other hand in the group of children on solid food diet (the majority eat solid food exclusively and one had bottle feeding) there were similar percentages of positives among children over 2 years in comparison with children under 2 years (59.8% *vs* 64%).

### 2. In-hospital children from an urban area (Aires de Menezes Hospital)

#### 2.1 Helminthes, protozoan and *Enterocytozoon bieneusi*

The socio-demographical and clinical data from in-hospital children enrolled in this study, and the results obtained are presented in Tables 1, 2 and 3.

Children hospitalized at Aires de Menezes Hospital ranged from 10 days to 10 years old with a median age of 1 year. Of the 214 children enrolled in the study, 57.5% (123/214) were male and 42.5% (91/214) were female.

Of the 214 in-hospital children studied, 60.3% (129/214) presented diarrhea, 57.9% (124/214) had gastrointestinal complaints and 51.4% (110/214) displayed fever.

The primary sources of drinking water reported in the questionnaire were piped water (36.9%; 79/214) and wells (14.0%; 30/214). Only 33.6% (72/214) had public sewer and 29.4% (63/214) had septic tank at home. About 64.2% (116/214) reported an indoor toilet.

Among the group of in-hospital children, 40.7% were on solid food diet, 25.2% were both breast fed and on solid food and 16.4% (87/214) were just breast fed only. In addition, 9.8% (21/214) of the children were on breast milk, bottle milk and solid food, 7.0% (15/214) were bottle fed and on solid food and about 0.9% (2/214) are just bottle fed.

Concerning food preservation 33.2% (71/214) had refrigerators at home. About 58.4% (125/214) of the children had domestic animals.

Among the 214 in-hospital children, 29.4% (63/214) presented intestinal parasites in fecal samples. In 22.4% (48/214) and 7.0% (15/214) of the parasitized, respectively, one or more species concurrently were detected, by microscopy and/or PCR methods (Table 2). As observed in children from Agostinho Neto village, *A. lumbricoides* (10.3%; 22/214) was also the most prevalent parasite in in-hospital children. The helminthes *T. trichiura* (6.5%; 14/214), *Schistosoma intercalatum* (0.9%; 2/214), *Ancylostoma* spp. (0.5%; 1/214) and the protozoa *Cryptosporidium* spp. (4.2%; 9/214), *G. duodenalis* (1.9%:4/214) and *E. coli* (0.5%; 1/214) were identified by microscopy.

Nineteen samples (8.9%; 19/214) from in-hospital children were *Cryptosporidium* positive using 18S PCR and gp60 PCR (only nine samples were positive by both microscopy and PCR methods), and all samples were successfully subtyped at the gp60 locus. Sequence analysis of the gp60 locus identified *C. hominis* (6.5%; 14/214) and *C. parvum* (2.3%; 5/214).

Two subtype families were identified within *C. hominis*: Ia and Ie. Altogether, four subtypes were observed: IaA27R3 (35.7%; 5/14), IaA23R3 (14.3%; 2/14), IeA11G3T3 (28.6%; 4/14) and IeA11G3T3R1 (21.4%; 3/14). Infections with *C. parvum* belonged to two subtype families: IIa and IId, and the subtype family IId was the most commonly found. Four subtypes were identified within the subtype families: IIaA16G2R1 (20.0%; 1/5), IIaA15G2R1 (20.0%; 1/5), IIdA26G1 (40.0%; 2/5) and IIdA21G1a (20.0%; 1/5).

*G. duodenalis* and *E. bieneusi* were also identified by PCR in 0.5% (1/214) (the sample was also positive by microscopy) and 8.9% (19/214) of the in-hospital children, respectively (Table 3). Sequence analysis of TPI locus identified Assemblage B in the only *G. duodenalis* positive sample. Based on PCR analysis of the *E. bieneusi* ITS region, four known genotypes in th GenBank database were identified: K (52.6%; 10/19), D 26.4%; 5/19), A (10.5%; 2/19) and KIN1 (10.5%; 2/19)

Among the children found to be positive for helminthes, protozoa and *E. bieneusi* more than 50% reported diarrhea and/or gastrointestinal complaints.

#### 2.2. Intestinal parasites and risk factors

No significant association between the variables analyzed (age, gender, symptoms, source of drinking water, sanitation, food preservation and domestic animals) and the presence of intestinal parasites was observed in children from the Aires de Menezes Hospital. However, a higher number of children infected with intestinal parasites were observed among those who were on solid food diet (exclusively or had combined feeding) (21.2%; 38/179) than those who were breastfed (5.7%; 2/35) (χ2 = 4,637, *P* = 0.031).

As noticed for the children of the village, in the group of breastfed (exclusively) all children were more than 2 years old and in only two children (5,7%) the occurrence of intestinal parasites was detected by microscopy. In the group of children on solid food diet (exclusively or had combined feeding) were detected a higher percentage of intestinal parasite-positives (43,4%) among children over 2 years old in comparison with children under 2 years (11,9%). Moreover, this latter association is statistically significant (χ2 = 23.125, P≤0.001)

### 3. Children from Agostinho Neto village *vs* in-pediatric Aires de Menezes Hospital population

Among the total of 348 children enrolled in this study the occurrence of fever (51.4%; 110/214) (χ2 = 67,782, *P*≤0.001), diarrhea (60.3%; 129/214) (χ2 = 74,124, *P*≤0.001) and gastrointestinal complaints (57.9%; 124/214) (χ2 = 36,940, *P*≤0.001) was significantly higher in children from Aires de Menezes Hospital. The presence of public sewer (33.6%; 72/214) and piped water (36.9%; 79/214) was significantly more common at houses from the in-hospital children than in the other population studied. Also, domestic animals were reported to be more frequent among in-hospital children.

The occurrence of helminthes and protozoa was more frequent among children from Agostinho Neto village, 33.6% (45/134) and 29.1% (39/134) versus 12.6% (27/214) and 11.2% (24/214) in in–hospital children, respectively (helminthes: χ2 = 22,073, *P*≤0.001; protozoa: χ2 = 17,787, *P*≤0.001). *E. bieneusi* was more common among in-hospital children (8.9%; 19/214) than in children from the rural population (5.2%; 7/134) studied, but this difference was not statistically significant.

Children from Agostinho Neto village showed significantly higher probability to be infected by at least one species (32.1%; 43/134) or more species concurrently (20.1%; 27/134) than those from Aires de Menezes Hospital (22.4%; 48/214, 7.0%; 15/214) (χ2 = 21,662, *P*≤0.001).

The presence of *Cryptosporidium* spp. (microscopy and/or PCR) was significantly higher in children from the hospital than in children from the rural area (8.9%; 19/214 *vs* 0%; 0/134, χ2 = 12,584, *P*≤0.001). In contrast, the frequency of *G. duodenalis* (microscopy and/or PCR) was significantly higher in children in the Agostinho Neto village (18.7%; 25/134) than in in-hospital children (1.9%; 4/214) (χ2 = 30,401, *P*≤0.001).

## Discussion

Intestinal parasites once considered to be controllable in developed countries remain a major cause of morbidity and mortality worldwide [20]. Poor water supply and sanitation are two of the most critical factors associated with the spread of intestinal diseases in low-income countries. Most of the population living in these regions is comprised of people without sustainable access to safe drinking water and basic sanitation. The public health importance of intestinal pathogens as a major concern in most developing countries has been exacerbated by the co-occurrence of malnutrition, malaria and HIV/AIDS. In addition, reported infection rates may be underestimated, because most of the health institutions lack suitable diagnostic methods to detect low levels of parasites. Some diagnostic tools for specific intestinal parasites, especially for the newly emerging opportunistic pathogen *E. bieneusi* species, are not available in peripheral health institutions [20].

DRSTP is a low-income tropical country, where climatic and living conditions facilitate the transmission of many intestinal pathogens including helminthes, protozoa, and the emerging *E. bieneusi*, particularly in children. Children are naturally more susceptible to infections and generally have poorer personal hygiene habits, thus with a potential higher exposure to intestinal organisms.

The present study has shown that the prevalence of intestinal parasites is high among children in Agostinho Neto village (52.2%), a typical rural African community, and in-hospital pediatric population (29.4%) in São Tomé Island. Marked differences on monoparasitism and polyparasitism rates were observed between the two populations. Children from the rural community were significant more likely to be infected with single and multiple species of parasites than the in-hospital population. The higher frequency of single or multiple-pathogen infection in children from Agostinho Neto village may possibly be linked to specific characteristics of this population. Children living in the rural community can be subject to a higher degree of environmental exposure to potential sources of infection (e.g. contaminated water, farm animals and wildlife), may have distinct behavioral habits (e.g. the present population included older children with more autonomy) and poorer hygiene practices and sanitation that may increase the risk of infection in comparison with the hospitalized children. Polyparasitism is likely the accumulated outcome of infection routes, host exposures, and susceptibility, as well as behavioral, sociological, and economic factors. Some studies have reported that polyparasitism may contribute more to morbidity than single-species infections. Also, multiple species infections may increase susceptibility to other infections, such as malaria. Consequently, efforts have to be made to better understand the consequences of the co-existence of parasites within the same host on the immunological responses to each species and, more importantly, whether such interactions affect susceptibility or clinical outcome of other pathogens [21], [22].

The overall frequency of helminthes (33.6% *vs* 12.6%) and protozoa (29.1% *vs* 11.2%) were significantly higher in Agostinho Neto village's children than in-hospital children. The difference observed between the two populations is probably also associated with the antiparasitic therapy (albendazole) campaign targeting children from the urban community in the Aires de Menezes Hospital, which occurred 2–3 months before this study. However, this may only explain the lower occurrence of helminthes and *Giardia* since the antiparasitic drugs used are almost ineffective on the other protozoa.

Although several common helminthes were identified in fecal samples from both groups, *A. lumbricoides* (27.6% *vs* 10.3%) and *T. trichiura* (6.5% *vs* 4.5%) were the most prevalent. The infection rate of these parasites is within the range described in other studies. Nevertheless, caution should be taken when interpreting differences in infection rates using data that could have been obtained using different methodologies and/or from specific groups of population. Pampiglione et al. in 1987 reported higher prevalence of *A. lumbricoides* (87.7%) and *T. trichiura* (64.3%) in children and adults from São Tomé and Principe. The same authors also identified 4.6% of Ancylostomidae, 6.8% of *S. stercoralis* and 0.2% of *Hymenolepis* [15]. More recently Belo et al. in 2005 identified *A. lumbricoides*, *T. trichiura* and Ancylostomidae in 70.8%, 68.5% and 4.6%, respectively in schoolchildren, from São Tomé [23]. The overall prevalence of protozoan *G. duodenalis* and *Cryptosporidium* spp. was statistically different between the two populations analyzed: *G. duodenalis* was detected more often in the rural region (7.5% vs 1.9%) and *Cryptosporidium* spp. were detected more often in-hospital children (8.9% *vs* 0%) (Table 3). The prevalence of these parasites in both populations is lower than the prevalence described in some studies on African pediatric populations. Epidemiological studies reported a high incidence of parasite infections, including *Cryptosporidium* spp. and *G. duodenalis* during the rainy season as compared to the hot and dry season [24]–[28]. As the fecal samples collected in this study were collected during the dry season (July-August), this could be the reason for the low presence and/or absence of these intestinal parasites among the two children populations analyzed. Both *Cryptosporidium* spp. and *G. duodenalis* are waterborne and therefore a high prevalence is expected in the wet season.

The significant association between *Cryptosporidium* infection and in-pediatric hospital population may be linked to: a) children from the hospital have higher physical debility due to disease responsible for hospitalization. In addition, in this study the symptoms (fever, diarrhea and gastrointestinal complaints) were more commonly reported among in-patients group; b) lower median age observed among in-patients (1 year old) in comparison with the median age of children from the rural area (4 years old). Children younger than 2 years of age are frequently infected in community and hospital settings in developing countries [25], [29]. Both reasons stated may predispose in-hospital population to be more vulnerable to the infection by an opportunistic parasite as *Cryptosporidium* spp.

The significant association between *G. duodenalis* infection and children from the rural region may be related to any behavioral habit and/or source of environmental infection among this area. For instance, consumption of unsafe water clearly represents a significant risk for giardiasis. However, the contamination of such water supplies may result from humans, farm animals, and wildlife. [30]. More studies are needed to track *G. duodenalis* source(s) of infections among this region. The majority of children from Agostinho Neto village seemed to be asymptomatic. Out of a total of 25 *G. duodenalis* positive-cases only two children reported diarrhea, and seven had gastrointestinal complaints (but were also co-infected with other intestinal organisms). Heresi and Cleary suggested that *Giardia* were part of the natural intestinal flora of people in tropical countries [31]; however, they are likely to be present as a result of poor environmental sanitation. Although it was not possible to assess the implications of *G. duodenalis* infection in the health of these children (e.g. evaluate their nutritional status), chronic infections are frequently associated with impaired child growth emphasizing the importance of these results to implement monitoring and treatment of giardiasis among children from this endemic area.

In this study, *E. bieneusi* carriage was higher in-hospital children (8.9%) than in children from the rural community (5.2%). Both rates determined are similar to those found in previous studies on African populations (children and/or adults): Democratic Republic of Congo (1.7%) [32], Gabon (3.0%) [33] Cameroon (5.2%) [34], Uganda (6.6%) [35], Nigeria (9.3%) [36], Niger (10.5%) [37]. *E. bieneusi* were more common among in-hospital population (of the total of 19 *E. bieneusi*-positives, 16 ranged from 0–2 years) than in children from Agostinho Neto village (the seven *E. bieneusi*-positives age ranged from 2–10 years) but this difference was not statistically significant. However, the reasons specified above to justify the higher number of cases of *Cryptosporidium* spp. infection among the in-hospital population can also be applied for *E. bieneusi* since this species is also considered an opportunistic organism and may affect younger and sick children than the apparently healthy children from the rural community.

*Cryptosporidium hominis* and *C. parvum* were the only species identified among in-hospital children. The presence of a higher rate of *C. hominis* (6.5%) than *C. parvum* (2.3%) is in accordance with previous studies in Africa and other developing countries, where 79–90% of infections are caused by *C. hominis*. [38]. Variations in the distribution of *Cryptosporidium* species in humans are considered an indication of differences in infection sources [18]. The predominance of *C. hominis* has also been reported in other paediatric populations in Africa, such as in South Africa [39]–[41] Malawi [42], Kenya [43], Uganda [35] and Nigeria [44], indicating that anthroponotic transmission may play a major role in the epidemiology of *Cryptosporidium* in this continent.

Both *C. hominis* Ia and Ie subtype families characterized in this study are included in the group of the four most commons subtype families (Ia, Ib, Id and Ie) that are usually observed in children in developing countries [9], [41], [44]. Infections with the Ia and Ie subtype families were more likely asymptomatic [17]. However, in the present study, 10 of the 14 *C. hominis*-positive in-hospital children had diarrhea, although the diarrhea could have been caused by other concomitant pathogens.

The *C. hominis* Ia subtypes identified, IaA27R3 (35.7%) and IaA23R3 (14.3%), have been previously described in GenBank Database. The *C. hominis* Ia subtype family observed in this study has been reported as one of the predominant subtype families in humans [45], including Nigeria [36], [44], [46], [47]. The *C. hominis* Ie subtypes identified, IeA11G3T3 (28.6%) and IeA11G3T3R1 (21.4%) have been reported in human infections from other african low-middle income countries, such as Nigeria [44], [46] and South Africa [41]. Similarly, to our results, in most low-middle income countries, humans with the subtype family Ie are mostly infected with subtype IeA11G3T3, with few exceptions for Jamaica and China where IeA12G3T3 has been detected [48], [49]

Although the anthroponotic IIc subtypes of *C. parvum* are responsible for most human infections in low-middle income countries [38], [39], [42], [50] only the zoonotic IIa and IId subtypes, IIaA16G2R1 (20.0%), IIaA15G2R1 (20.0%), IIdA26G1 (40.0%) and IIdA21G1a (20.0%) were identified in the study. In the African continent, Molloy et al., reported IIa *C. parvum* subtypes (IIaA15G2R1and IIaA16G1R1) in two children from rural Nigeria [44], and Adamu et al. reported IIa *C. parvum* subtypes (IIaA15G2R1, IIaA16G2R1 and IIaA16G1R1) in 12 children in Ethiopia [51]. The IIa and, to a less degree, IId are also common *C. parvum* subtype families in European countries [9], [52], and the subtype IIa has been detected in both humans and ruminants, for instance in Iran and other Mideast countries [53].The finding of IIa and IId subtypes families in this study may suggest that *C. parvum* in children in São Tomé island is likely to be of zoonotic origin. However the *C. parvum* IIa subtype family usually described as zoonotic was reported previously as being associated to anthroponotic transmission in developing countries [9]. Similarly to the present study, iIqbal et al. reported Ia and Ie *C. hominis* and IIa and IId *C. parvum* as the more frequent subtype families among children from Kuwait [54].

Sequence analysis of TPI locus identified Assemblages A (60%) and B (40%) of *G. duodenalis* among children from Agostinho Neto village, and Assemblage B (100%) in-pediatric hospital population. Studies on genetic characterization of *Giardia* in humans in the sub-Saharan Africa are scarce [55], [56]. East Africa is considered to be the most endemic regions for *G. duodenalis* [57]. One of these studies was performed in rural western Uganda and *G. duodenalis* typing showed that the distribution between assemblage A and B was 53% and 47%, respectively [56]. More recently, Ankarklev et al. reported a predominance of Assemblage B, followed by assemblage AII and by mixed assemblage infections of *G. duodenalis* among young children in Uganda [58]. In Rwanda, 85.9% of *G. duodenalis*-positive samples isolated from children were assemblage B and 12.7% were assemblage A2, in addition to one assemblage A1 and one mixed assemblage A+B [59]. In rural and in-hospital children in São Tomé island, anthroponotic assemblages A and B predominate, indicating person-to-person transmission plays an important role in giardiasis epidemiology. This is supported also by the absence of animal-specific assemblages of *G. duodenalis*, such as C and D, which are common in dogs, and E, F, G, which are common in livestock, cats, and rodents, respectively [30].

The transmission of *E. bieneusi* in São Tomé is less clear due to the lack of data concerning this organism in the country. The majority of *E. bieneusi* found in this study belong to the group that infects both humans and animals. Within this group, genotype Type IV was the dominant one (in both groups of the children studied). D and A genotypes were found only in-pediatric hospital population. Both Type IV and D are well known zoonotic *E. bieneusi* genotypes, having been reported in humans, macaque, baboons, cattle, pigs, dogs, and some wild mammals. In contrast, genotype A is mostly an anthroponotic genotype. Both genotypes KIN1 (identified among children from Agostinho Neto Village and in-hospital population) and KIN3 (in children from Agostinho Neto Village) are within the ITS *E. bieneusi* genotypes group identified exclusively in humans until now. Studies carried in African countries as Uganda (Type IV) [60], Cameroon (A, D and Type IV) [33], [34], Niger (A, D and Type IV) [37], Gabon (A, D and Type IV) [33], Malawi (D and Type IV) [61], Democratic Republic of Congo (D, KIN1 and KIN3) [32] and Nigeria (D and Type IV) [36], [62] reported a common occurrence of these genotypes in humans. These results suggest that both antroponotic and zoonotic transmission might be responsible for *E. bieneusi* infection in the children studied. However, more systematic collection of epidemiological data and sampling of animals, drinking water, and fresh produce are needed to clarify the source of *E. bieneusi* in DRSTP children.

Both the overall detection of intestinal parasites and the cases of monoparasitism and/or polyparasitism identified by microscopy among children from Agostinho Neto Village were significantly associated with the presence of septic tank next to home. Also, significant associations between *G. duodenalis* PCR-positive and children that reported having well or septic tank near the house were observed. Thus, risk of exposure to intestinal pathogens identified as a result of inadequate water and sanitation is high among this rural population. Reducing the risks to which children from this region are exposed through unsafe water or adequate sanitation is particularly important given the large disease burden attributable to these issues, especially in children.

The significant association between the presence of intestinal microorganisms detected by microscopy and children that consume solid food (exclusively or have combined feeding) from both populations analyzed suggest that those parasites can have foodborne origin. However, it must be taken into account that the start of intake of solid food is associated to an increased mobility of the child, changes on behavioral and hygienic habits and therefore to an increased contact with the environment and other potential risk factors. Although no definite conclusions can be drawn as seen in other studies [63], [64], data from this study suggest that breastfeeding is highly protective against intestinal diseases. For instance, protection from *G. duodenalis* infection by breast milk has previously been reported [65]. Taking into account the high endemicity of *G. duodenalis* in the study areas it seems plausible that protective antibodies [66] are transmitted by the breast-milk of seropositive mothers. In addition, lactoferrin and leukocytes in breast milk could be involved in protection of the younger children under breastfeeding [67].

Clinical features of these intestinal parasites are indistinguishable from one another, ranging from asymptomatic, to severe watery diarrhea, fever, weight loss, and possible extraintestinal manifestations. Although the presence of fever, diarrhea and gastrointestinal complaints were more commonly reported among in-pediatric children no statistically significant association were observed between the presence of symptoms and any of intestinal pathogens identified. Thus, the majority of children analyzed seem to be asymptomatic in accordance with reports by others. Nevertheless, asymptomatic carriage may cause severe long-term consequences for the child's health. Beyond that these children play a crucial role in the transmission of theses parasites to other susceptible hosts, working as continuous reservoir for these parasites.

This is an important study because it provides updated data on the occurrence of several intestinal pathogens in two different communities in São Tome Island. To our knowledge, this is the first record on genetic characterization of *Cryptosporidium* spp., *G. duodenalis* and *E. bieneusi* in children from this African country. Data from the present study have suggested a major role of anthroponotic transmission in the epidemiology of cryptosporidiosis and giardiasis. Both anthroponotic and zoonotic transmission were probably involved in microsporidiosis. Further molecular epidemiological studies enrolling domestic and wild animals, water and other potential sources of environmental infection in well-defined endemic locations are required to clarify de epidemiology of these infectious diseases in this tropical country.

While improving sustainable access to safe drinking water and basic sanitation facilities is a key target, the impact of both personal and domestic hygiene behaviours should not be neglected. Educational campaigns (e.g., promote handwashing, lectures on measures of prevention and diseases control and exclusively breastfeeding) among children and their caretakers have been shown to be an effective tool in reducing the risk of infection and reinfection in children improving children's health from these communities [63], [68]–[71]. These campaigns should be implemented for routine in this endemic region. Changing the population behavior is a long-term process, since so many times it is linked to socio-cultural habits but with targeted training meaningful positive improvement can be achieved.

Almost half of DRSTP population may be considered vulnerable to infections, namely by parasites, since nearly 44% has between 0 and 14 years old (2012), and is estimated that each year about 13.1% of children under the age of 5 years (2009) is underweight due to malnutrition [11]. Moreover, a large majority do not have access to sustainable drinking water and proper sanitation. In summary, the specific scenario observed in this endemic region highlights the significance of the results obtained in the present study and suggests that it is crucial to strengthen surveillance on intestinal pathogens. The results of this study were reported to the health authorities of S. Tomé e Príncipe in order to implement the appropriate treatment measures among this population and the authors expect that this data have contributed to the improvement of children's health from these communities. The synergistic effect of policies targeting both improvements of hygiene and health conditions of the population by the competent authorities of DRSTP, will enhance the successful outcomes with future programs for prevention and eradication of intestinal infections, one of the major health risks to children.

## References

[1] Adeoye GO, Osatemi CO, Otenia O, Onyemekeihia SO (2007) Epidemiological studies of intestinal helminthes and malaria among children in Lagos, Nigeria. Pakistan. J Biol Sciences 10 (13): 2208–2212. 10.3923/pjbs.2007.2208.2212 19070183

[2] Samuel L, Stanley Jr, Sharon L (2001) Reed: microbes and microbial toxins: paradigms for microbial-mucosal interactions. VI *Entamoeba histolytica*: parasite host interactions. Am J Physiol Gastrointest Liver Physiol 280: 1049–1054. 10.1152/ajpgi.2001.280.6.G1049 11352795

[3] de Silva NR, Brooker S, Hotez PJ, Montresor A, Engels D, et al (2003) Soil transmitted helminth infections: updating the global picture. Trends Parasitol 19: 547–551. 14642761 10.1016/j.pt.2003.10.002

[4] Ali SA, Hill DR (2003) *Giardia intestinalis* . Curr Opin Infect Dis 16: 453–460. 14501998 10.1097/00001432-200310000-00012

[5] Xiao L, Fayer R (2008) Molecular characterization of species and genotypes of *Cryptosporidium* and *Giardia* and assessment of zoonotic transmission. Int J Parasitol 38: 1239–1255. 18479685 10.1016/j.ijpara.2008.03.006

[6] Kotloff KL, Nataro JP, Blackwelder WC, Nasrin D, Farag TH, et al (2013) Burden and aetiology of diarrhoeal disease in infants and young children in developing countries (the Global Enteric Multicenter Study, GEMS): a prospective, case-control study. Lancet 382(9888): 209–222. 23680352 10.1016/S0140-6736(13)60844-2

[7] Lobo ML, Xiao L, Antunes F, Matos O (2012) Microsporidia as emerging pathogens and the implication for public health: a 10-year study on HIV-positive and -negative patients. Int J Parasitol 42(2): 197–205. 22265899 10.1016/j.ijpara.2011.12.002

[8] Savioli L, Smith H, Thompson A (2006) *Giardia* and *Cryptosporidium* join the ‘Neglected Diseases Initiative’. Trends Parasitol 22: 203–208. 16545611 10.1016/j.pt.2006.02.015

[9] Xiao L (2010) Molecular epidemiology of cryptosporidiosis: an update. Exp. Parasitol 124: 80–89. 10.1016/j.exppara.2009.03.018 19358845

[10] Nyarango RM, Aloo PA, Kabiru EW, Nyanchongi BO (2008) The risk of pathogenic intestinal parasite infections in Kisii Municipalità, Kenya. BMC Public Health 8: 237–242. 18620608 10.1186/1471-2458-8-237 PMC2478685

[11] Central Intelligence Agency. Publications. Available: https://www.cia.gov/library/publications/the-world-factbook/geos/tp.html.

[12] The World Bank. Countries. Available: http://www.worldbank.org/en/country/saotome/overview.

[13] Azam SA, Rahman Bhuiyan MM, Zaforullah Choudhury M, Ali Miah K (2007) Intestinal parasites and sanitary practices among the rural children. *TAJ* 20 (1): 1–5.

[14] Ripert C, Neves I, Appriou M, Tribouley J, Tribouley-Duret J, et al (1996) Epidemiology of certain endemic parasitic diseases in the town of Guadalupe (Republic of Sao Tome and Principe) I. Schistosomiasis *intercalatum* and intestinal worms. Bull Soc Pathol Exot 89(4): 252–258. 9053044

[15] Pampiglione S, Visconti S, Pezzino G (1987) Human intestinal parasites in Subsaharan Africa. II. Sao Tomé and Principe. Parassitologia 29(1): 15–25. 3508506

[16] Lobo ML, Xiao L, Cama V, Magalhães N, Antunes F, et al (2006) Identification of potentially human-pathogenic *Enterocytozoon bieneusi* genotypes in various birds. Appl Environ Microbiol 72(11): 7380–382. 16936045 10.1128/AEM.01394-06 PMC1636141

[17] Cama VA, Ross JM, Crawford S, Kawai V, Chavez-Valdez R, et al (2007) Differences in clinical manifestations among *Cryptosporidium* species and subtypes in HIV-infected persons. J Infect Dis 196(5): 684–91. 17674309 10.1086/519842

[18] Sulaiman IM, Hira PR, Zhou L, Al-Ali FM, Al-Shelahi FA, et al (2005) Unique endemicity of cryptosporidiosis in children in Kuwait. J Clin Microbiol 43: 2805–2809. 15956401 10.1128/JCM.43.6.2805-2809.2005 PMC1151898

[19] Sulaiman IM, Fayer R, Bern C, Gilman RH, Trout JM, et al (2003) Triosephosphate isomerase gene characterization and potential zoonotic transmission of *Giardia duodenalis* . Emerg Infect Dis 9(11): 1444–1452. 14718089 10.3201/eid0911.030084 PMC3035538

[20] Alemu A, Shiferaw Y, Getnet G, Yalew A, Addis Z (2011) Opportunistic and other intestinal parasites among HIV/AIDS patients attending Gambi higher clinic in Bahir Dar city, North West Ethiopia. Asian Pac J Trop Med 4(8): 661–665. 21914548 10.1016/S1995-7645(11)60168-5

[21] Supali T, Verweij JJ, Wiria AE, Djuardi Y, Hamid F, et al (2010) Polyparasitism and its impact on the immune system. Int J Parasitol 40: 1171–1176. 20580905 10.1016/j.ijpara.2010.05.003

[22] Lustigman S, Prichard RK, Gazzinelli A, Grant WN, Boatin BA, et al (2012) A research agenda for helminth diseases of humans: the problem of helminthiases. PLoS Negl Trop Dis 6(4): e1582. 22545164 10.1371/journal.pntd.0001582 PMC3335854

[23] Belo S, Rompão H, Gonçalves L, Grácio MA (2005) Prevalence, behavioural and social factors associated with *Schistosoma intercalatum* and geohelminth infections in São Tomé and Principe. Parassitologia 47(2): 227–231. 16252477

[24] Moodley D, Jackson TF, Gathiram V, van den Ende J (1991) *Cryptosporidium* infections in children in Durban. Seasonal variation, age distribution and disease status. S Afr Med J 79(6): 295–297. 2017736

[25] Bern C, Hernandez B, Lopez MB, Arrowood MJ, De Merida AM, et al (2000) The contrasting epidemiology of *Cyclospora* and *Cryptosporidium* among outpatients in Guatemala. Am J Trop Med Hyg 63(5,6): 231–235. 11421369

[26] Perch M, Sodemann M, Jakobsen MS, Valentiner-Branth P, Steinsland H, et al (2001) Seven years' experience with *Cryptosporidium parvum* in Guinea-Bissau, West Africa. Ann Trop Paediatr 21(4): 313–318. 11732149 10.1080/07430170120093490

[27] Tumwine JK, Kekitiinwa A, Nabukeera N, Akiyoshi DE, Rich SM, et al (2003) *Cryptosporidium parvum* in children with diarrhea in Mulago Hospital, Kampala, Uganda. Am J Trop Med Hyg 68(6): 710–715. 12887032

[28] Siwila J, Phiri IG, Enemark HL, Nchito M, Olsen A (2011) Seasonal prevalence and incidence of *Cryptosporidium* spp. and *Giardia duodenalis* and associated diarrhoea in children attending pre-school in Kafue, Zambia. Trans R Soc Trop Med Hyg 105(2): 102–108. 21093003 10.1016/j.trstmh.2010.10.004

[29] Cama VA, Bern C, Roberts J, Cabrera L, Sterling CR, et al (2008) *Cryptosporidium* species and subtypes and clinical manifestations in children. Peru Emerg Infect Dis 14(10): 1567–1574. 18826821 10.3201/eid1410.071273 PMC2609889

[30] Feng Y, Xiao L (2011) Zoonotic potential and molecular epidemiology of Giardia and giardiasis. Clin Microbiol Rev 24: 110–140. 21233509 10.1128/CMR.00033-10 PMC3021202

[31] Heresi G, Cleary TG (1997) *Giardia* . Pediatr Rev 18(7): 243–247. 9203832 10.1542/pir.18-7-243

[32] Wumba R, Longo-Mbenza B, Mandina M, Odio WT, Biligui S, et al (2010) Intestinal parasites infections in hospitalized AIDS patients in Kinshasa, Democratic Republic of Congo. Parasite 17: 321–328. 21275238 10.1051/parasite/2010174321

[33] Breton J, Bart-Delabesse E, Biligui S, Carbone A, Seiller X, et al (2007) New highly divergent rRNA sequence among biodiverse genotypes of *Enterocytozoon bieneusi* strains isolated from humans in Gabon and Cameroon. J Clin Microbiol 45: 2580–2589. 17537939 10.1128/JCM.02554-06 PMC1951207

[34] Sarfati C, Bourgeois A, Menotti J, Liegeois F, Moyou-Somo R, et al (2006) Prevalence of intestinal parasites including microsporidia in human immunodeficiency virus-infected adults in Cameroon: a cross-sectional study. Am J Trop Med Hyg 74: 162–164. 16407362

[35] Tumwine JK, Kekitiinwa A, Bakeera-Kitaka S, Ndeezi G, Downing R, et al (2005) Cryptosporidiosis and microsporidiosis in ugandan children with persistent diarrhea with and without concurrent infection with the human immunodeficiency virus. Am J Trop Med Hyg 73(5): 921–925. 16282304

[36] Ayinmode AB, Ojuromi OT, Xiao L (2011) Molecular identification of *Enterocytozoon bieneusi* isolates from Nigerian children. J Parasitol Res 129542. 10.1155/2011/129542 PMC3216387 22132304

[37] Espern A, Morio F, Miegeville M, Illa H, Abdoulaye M, et al (2007) Molecular study of microsporidiosis due to *Enterocytozoon bieneusi* and *Encephalitozoon intestinalis* among human immunodeficiency virus-infected patients from two geographical areas: Niamey, Niger, and Hanoi, Vietnam. J Clin Microbiol 45: 2999–3002. 17634305 10.1128/JCM.00684-07 PMC2045311

[38] Xiao L, Feng Y (2008) Zoonotic cryptosporidiosis. FEMS Immunol Med Microbiol 52: 309–323. 18205803 10.1111/j.1574-695X.2008.00377.x

[39] Leav BA, Mackay MR, Anyanwu A, Connor RM, Cevallos AM, et al (2002) Analysis of sequence diversity at the highly polymorphic Cpgp40/15 locus among *Cryptosporidium* isolates from human immunodeficiency virus-infected children in South Africa. Infect Immun 70: 3881–3890. 12065532 10.1128/IAI.70.7.3881-3890.2002 PMC128099

[40] Samie A, Bessong PO, Obi CL, Sevilleja JE, Stroup S, et al (2006) *Cryptosporidium* species: Preliminary descriptions of the prevalence and genotype distribution among school children and hospital patients in the Venda region, Limpopo Province, South Africa. Exp Parasitol 114: 314–322. 16806189 10.1016/j.exppara.2006.04.007

[41] Abu Samra N, Thompson PN, Jori F, Frean J, Poonsamy B, et al (2013) Genetic characterization of *Cryptosporidium* spp. in diarrhoeic children from four provinces in South Africa. Zoonoses Public Health 60(2): 154–159. 22712773 10.1111/j.1863-2378.2012.01507.x

[42] Peng MM, Meshnick SR, Cunliffe NA, Thindwa BDM, Hart CA, et al (2003) Molecular epidemiology of Cryptosporidiosis in children in Malawi. J Eukaryot Microbiol 50: 557–559. 14736161 10.1111/j.1550-7408.2003.tb00628.x

[43] Gatei W, Wamae C, Mbae C, Waruru A, Mulinge E, et al (2006) Cryptosporidiosis: prevalence, genotype analysis, and symptoms associated with infections in children in Kenya. Am J Trop Med Hyg 75: 78–82. 16837712

[44] Molloy SF, Smith HV, Kirwan P, Nichols RA, Asaolu SO, et al (2010) Identification of a high diversity of *Cryptosporidium* species genotypes and subtypes in a pediatric population in Nigeria. The American Journal of Tropical Medicine and Hygiene 82 (4): 608–613. 20348508 10.4269/ajtmh.2010.09-0624 PMC2844578

[45] Xiao L, Ryan UM (2004) Cryptosporidiosis: an update in molecular epidemiology. Current Opinion in Infectious Diseases 17: 483–490. 15353969 10.1097/00001432-200410000-00014

[46] Akinbo FO, Okaka CE, Omoregie R, Dearen T, Leon ET, et al (2010) Molecular characterization of *Cryptosporidium* spp. in HIV-infected persons in Benin City, Edo State, Nigeria. Fooyin J Health Sci 2: 85–89.

[47] Maikai BV (2012) Molecular characterizations of *Cryptosporidium*, *Giardia*, and *Enterocytozoon* in humans in Kaduna State, Nigeria. Exp Parasitol 131: 452–456. 22664352 10.1016/j.exppara.2012.05.011

[48] Gatei W, Barrett D, Lindo JF, Eldemire-Shearer D, Cama V, et al (2008) Unique *Cryptosporidium* population in HIV-infected persons, Jamaica. Emerg Infect Dis 14(5): 841–843. 18439378 10.3201/eid1405.071277 PMC2600223

[49] Feng Y, Li N, Duan L, Xiao L (2009) *Cryptosporidium* genotype and subtype distribution in raw wastewater in Shanghai, China: evidence for possible unique Cryptosporidium hominis transmission. J Clin Microbiol 47(1): 153–157. 19005143 10.1128/JCM.01777-08 PMC2620847

[50] Akiyoshi DE, Tumwine JK, Bakeera-Kitaka S, Tzipori S (2006) Subtype analysis of *Cryptosporidium* isolates from children in Uganda. J Parasitol 92: 1097–1100. 17152957 10.1645/GE-843R.1

[51] Adamu H, Petros B, Hailu A, Petry F (2010) Molecular characterization of *Cryptosporidium* isolates from humans in Ethiopia. Acta Trop 115: 77–83. 20206592 10.1016/j.actatropica.2010.02.003

[52] Alves M, Xiao L, Antunes F, Matos O (2006) Distribution of *Cryptosporidium* subtypes in humans and domestic and wild ruminants in Portugal. Parasitology Research 99: 287–292. 16552512 10.1007/s00436-006-0164-5

[53] Nazemalhosseini-Mojarad E, Feng Y, Xiao L (2012) The importance of subtype analysis of *Cryptosporidium* spp. in epidemiological investigations of human cryptosporidiosis in Iran and other Mideast countries. Gastroenterology and Hepatology From Bed to Bench 5: 67–70. 24834202 PMC4017458

[54] Iqbal J, Khalid N, Hira PR (2011) Cryptosporidiosis in Kuwaiti children: association of clinical characteristics with *Cryptosporidium* species and subtypes. J Med Microbiol 60(Pt 5): 647–652. 21233297 10.1099/jmm.0.028001-0

[55] Gelanew T, Lalle M, Hailu A, Pozio E, Caccio SM (2007) Molecular characterization of human isolates of *Giardia duodenalis* from Ethiopia. Acta Trop 102: 92–99. 17498637 10.1016/j.actatropica.2007.04.003

[56] Johnston AR, Gillespie TR, Rwego IB, McLachlan TL, Kent AD, et al (2010) Molecular epidemiology of cross-species *Giardia duodenalis* transmission in western Uganda. PLoS Negl Trop Dis 4: e683. 20485494 10.1371/journal.pntd.0000683 PMC2867944

[57] Ekdahl K, Andersson Y (2005) Imported giardiasis: impact of international travel, immigration, and adoption. Am J Trop Med Hyg 72: 825–830. 15964971

[58] Ankarklev J, Hestvik E, Lebbad M, Lindh J, Kaddu-Mulindwa DH, et al (2012) Common coinfections of *Giardia intestinalis* and *Helicobacter pylori* in non-symptomatic Ugandan children. PLoS Negl Trop Dis 6(8): e1780. 22953010 10.1371/journal.pntd.0001780 PMC3429385

[59] Ignatius R, Gahutu JB, Klotz C, Steininger C, Shyirambere C, et al (2012) High prevalence of *Giardia duodenalis* Assemblage B infection and association with underweight in Rwandan children. PLoS Negl Trop Dis 6(6): e1677. 22720102 10.1371/journal.pntd.0001677 PMC3373622

[60] Tumwine JK, Kekitiinwa A, Nabukeera N, Akiyoshi DE, Buckholt MA, et al (2002) *Enterocytozoon bieneusi* among children with diarrhea attending Mulago Hospital in Uganda. Am J Trop Med Hyg 67: 299–303. 12408671 10.4269/ajtmh.2002.67.299

[61] ten Hove RJ, Van Lieshout L, Beadsworth MB, Perez MA, Spee K, et al (2009) Characterization of genotypes of *Enterocytozoon bieneusi* in immunosuppressed and immunocompetent patient groups. J Eukaryot Microbiol 56: 388–393. 19602086 10.1111/j.1550-7408.2009.00393.x

[62] Ojuromi OT, Izquierdo F, Fenoy S, Fagbenro-Beyioku A, Oyibo W, et al (2012) Identification and characterization of microsporidia from fecal samples of HIV-positive patients from Lagos, Nigeria. PLoS ONE 7: 4. 10.1371/journal.pone.0035239 PMC3322150 22496910

[63] Bhandari N, Bahl R, Mazumdar S, Martines J, Black RE, et al (2003) Effect of community-based promotion of exclusive breastfeeding on diarrheal illness and growth: a cluster randomised controlled trial. Lancet 361: 1418–1423. 12727395 10.1016/S0140-6736(03)13134-0

[64] Moore SR, Lima AA, Conaway MR, Schorling JB, Soares AM, et al (2001) Early childhood diarrhoea and helminthiases associate with long-term linear growth faltering. Int J Epidemiol 30(6): 1457–1464. 11821364 10.1093/ije/30.6.1457

[65] Mahmud MA, Chappell CL, Hossain MM, Huang DB, Habib M, et al (2001) Impact of breast-feeding on *Giardia lamblia* infections in Bilbeis, Egypt. Am J Trop Med Hyg 65: 257–260. 11561714 10.4269/ajtmh.2001.65.257

[66] Tellez A, Winiecka-Krusnell J, Paniagua M, Linder E (2003) Antibodies in mother's milk protect children against giardiasis. Scand J Infect Dis 35: 322–325. 12875519 10.1080/00365540310008041

[67] Ochoa TJ, Chea-Woo E, Campos M, Pecho I, Prada A, et al (2008) Impact of lactoferrin supplementation on growth and prevalence of *Giardia* colonization in children. Clin Infect Dis 46: 1881–1883. 18462105 10.1086/588476 PMC2440650

[68] Luby SP, Agboatwalla M, Painter J, Altaf A, Billhimer WL, et al (2004) Effect of intensive handwashing promotion on childhood diarrhea in high-risk communities in Pakistan: a randomizedcontrolled trial. JAMA 291: 2547–2554. 15173145 10.1001/jama.291.21.2547

[69] Luby SP, Agboatwalla M, Feikin DR, Painter J, Billhimer W, et al (2005) Effect of handwashing on child health: a randomized controlled trial. Lancet 366: 225–233. 16023513 10.1016/S0140-6736(05)66912-7

[70] Gungoren B, Latipov R, Regallet G, Musabaev E (2007) Effect of hygiene promotion on the risk of reinfection rate of intestinal parasites in children in rural Uzbekistan. Trans R Soc Trop Med Hyg 101(6): 564–569. 17418321 10.1016/j.trstmh.2007.02.011

[71] Fouamno Kamga HL, Shey Nsagha D, Suh Atanga MB, Longdoh Njunda A, Nguedia Assob JC, et al (2011) The impact of health education on the prevalence of faecal-orally transmitted parasitic infections among school children in a rural community in Cameroon. Pan Afr Med J 8: 38. 22121446 10.4314/pamj.v8i1.71153 PMC3201601

